# Experiences and Interactions with the Healthcare System in Transgender and Non-Binary Patients in Austria: An Exploratory Cross-Sectional Study

**DOI:** 10.3390/ijerph18136895

**Published:** 2021-06-27

**Authors:** Lovro Markovic, Daragh T. McDermott, Sinisa Stefanac, Radhika Seiler-Ramadas, Darina Iabloncsik, Lee Smith, Lin Yang, Kathrin Kirchheiner, Richard Crevenna, Igor Grabovac

**Affiliations:** 1 Department of Social and Preventive Medicine, Centre for Public Health, Medical University of Vienna, 1090 Vienna, Austria; lovro.markovic@meduniwien.ac.at (L.M.); radhika.seiler-ramadas@meduniwien.ac.at (R.S.-R.); darina.iabloncsik@gmail.com (D.I.); igor.grabovac@meduniwien.ac.at (I.G.); 2 Department of Physical Medicine, Rehabilitation and Occupational Medicine, Medical University of Vienna, 1090 Vienna, Austria; richard.crevenna@meduniwien.ac.at; 3 NTU Psychology, School of Social Sciences, Nottingham Trent University, Nottingham NG1 4FQ, UK; daragh.mcdermott@ntu.ac.uk; 4 Institute of Outcomes Research, Centre for Medical Statistics, Informatics and Intelligent Systems, Medical University of Vienna, 1090 Vienna, Austria; 5 Cambridge Centre for Sport and Exercise Sciences, Anglia Ruskin University, Cambridge CB1 1PT, UK; lee.smith@aru.ac.uk; 6 Department of Cancer Epidemiology and Prevention Research, Cancer Care Alberta, Alberta Health Services, Calgary, AB T2S 3C3, Canada; lin.yang@albertahealthservices.ca; 7 Departments of Oncology and Community Health Sciences, Cumming School of Medicine, University of Calgary, Calgary, AB T2N 1N4, Canada; 8 Department of Radiation Oncology, Medical University of Vienna, 1090 Vienna, Austria; kathrin.kirchheiner@meduniwien.ac.at

**Keywords:** healthcare utilization, trans, transgender, non-binary, gender-nonconforming, physician–patient relationship, health behavior

## Abstract

Medical care of transgender and non-binary (TNB) patients if often a complex interdisciplinary effort involving a variety of healthcare workers (HCWs) and services. Physicians not only act as gatekeepers to routine or transitioning therapies but are also HCWs with the most intimate and time-intensive patient interaction, which influences TNB patients’ experiences and health behaviors and healthcare utilization. The aim of this study was to investigate the physician–patient relationship in a sample of TNB individuals within the Austrian healthcare system, and explore its associations with sociodemographic, health-, and identity-related characteristics. A cross-sectional study utilizing an 56-item online questionnaire, including the Patient-Doctor Relationship Questionnaire 9 (PDRQ-9), was carried out between June and October 2020. The study involved TNB individuals 18 or older, residing in Austria, and previously or currently undergoing medical transition. In total, 91 participants took part, of whom 33.0% and 25.3% self-identified as trans men and trans women, respectively, and 41.8% as non-binary. Among participants, 82.7% reported being in the process of medical transitioning, 58.1% perceived physicians as the most problematic HCWs, and 60.5% stated having never or rarely been taken seriously in medical settings. Non-binary participants showed significantly lower PDRQ-9 scores, reflecting a worse patient–physician relationship compared to trans male participants. TNB patients in Austria often report negative experiences based on their gender identity. Physicians should be aware of these interactions and reflect potentially harmful behavioral patterns in order to establish unbiased and trustful relations.

## 1. Introduction

Despite increasing social visibility and legal recognition, gender-minority individuals, including transgender (people whose gender identity differs from the normatively expected based on sex as assigned at birth) and non-binary (people whose gender identity does not match the binary conceptualization of gender) (TNB) people [1], still face social marginalization, discrimination, and numerous barriers in the healthcare system [2,3,4]. Experiencing transphobia (i.e., discrimination based on gender expression or identity that differs from the sex assigned at birth) has been a recognized issue in accessing healthcare services, including primary care as well as specialized secondary and tertiary care [2,4,5]. Barriers to accessing healthcare for TNB people are multifarious and range from subtle (such as ill-adapted environments and lack of knowledgeable personnel) to direct (such as denial of health care or abuse) [6,7]. In fact, results of a U.S.-based study reported that 19% of transgender identifying patients were denied medical care, with this proportion rising to 28% in transgender patients of color and 2% overall reported to have experienced physical violence in the physician’s office [8]. These reports are further compounded by robust evidence indicating higher prevalence of mental health problems in TNB people [9]. For example, studies report that depression is twice as prevalent in TNB youth compared to cisgender (i.e., those whose gender identity matches the gender assigned at birth) youth [9,10]. Moreover, 39% of TNB adults reported psychological distress over the past month and 40% reported suicide attempts over their lifetime [9]. Many of these mental health issues evolve as a result of experiencing prejudice and discrimination attributable to their gender identity (i.e., transphobia). Owen-Smith et al. reported that depression correlated with negative perceptions of community tolerance in TNB adults [11]. 

Mental health outcomes may be improved through services that provide empowering and affirming healthcare (i.e., provide respectful support to the patient’s self-identified gender identity, using appropriate chosen pronouns and names) [12]. For example, a study by Tucker et al. reported a decrease in suicidal ideation in trans U.S. army veteran patients who received affirming care compared to those who did not [13]. The results seem to be similar across age groups, as reports also showed that transgender prepubescent children (who have a binary gender identity that does not align with their sex as assigned at birth) who received affirming care and were allowed to present in their gender identity in everyday life had lower rates of depression and anxiety, which did not significantly differ from the two control groups included in this study (i.e., their siblings and age-matched non-transgender children) [14]. Furthermore, TNB adults who received medical care from healthcare providers that they considered to be affirming had lower levels of depression and suicidal ideation compared to TNB adults who reported not having a trans-affirmative healthcare provider [15]. Results from an Australian study showed that gender diverse Australians who felt respected by, and comfortable with, their general practitioners reported better mental health [16]. 

The importance of providing empowering and affirming healthcare for TNB patients has been outlined in various studies, and is confirmed by various prominent international guidelines and standards on transgender and non-binary care, yet studies of experiences with TNB patients show an immense lack of knowledge and information on TNB health from healthcare providers. In 2010, 50% of the 6450 surveyed transgender individuals reported having to educate their healthcare providers on various issues of transgender medical care, which is challenging and may be especially harmful in the patient–physician interaction [8]. This lack of knowledge and information is likely a result of training deficits on TNB health during medical education. For example, Chisolm-Starker et al. reported that even though 88% of emergency care staff provided care to transgender patients, 82.5% never received any formal training on the specific aspects of working with this population [17]. Lack of education combined with stigma contributes further to negative interactions with physicians and other professionals in healthcare settings [18]. Moreover, in addition to lack of training, there are results indicating that healthcare providers may hold bias towards LGBT patients that emanates from their lived experiences and personal political or religious views [19,20]. Even in healthcare providers that were receptive to providing LGBT competent care, some studies indicated that there are still enacted micro-aggressions that further maintain negative experiences of TNB people in healthcare. These often include nullifying LGBT experiences or their importance in the clinical setting, or through maintenance of stereotypes [21]. 

Unfavorable communication and interaction may include not understanding the needs of TNB patients, or not understanding the differences between constructs such as sex and gender or between sexual orientation and gender identity, which mostly stem from a lack of exposure to TNB people and education [7]. This may lead physicians to try to fit TNB patients into binary protocols and not provide affirmative care [22,23]. Negative experiences in the healthcare setting may not only be detrimental to physical and mental health, but also influence healthcare utilization, reduce quality of life, and increase rates of self-reported disability [18,24]. Moreover, considering the need to establish a medical precondition (i.e., diagnosis) in order to start medical transitioning, TNB patients may feel compelled to provide healthcare professionals with fictitious narratives in order to ensure the start of the medical transitioning process [2,25,26]. This constitutes yet another barrier for TNB patients in the healthcare setting and further underlines the importance of supportive patient–physician interactions. In Austria, initiating a medical transitioning process involves an assessment by three independent mental health professionals—a psychotherapist, a psychiatrist, and a psychologist—all of whom need to give an independent positive referral. These are summarized as a joint statement by a so-called “coordinator of care” as chosen by the patient, and may be any medical professional involved in the transitioning process [27]. Afterwards, patients may start with medical gender-affirming interventions as well as legal transitioning (i.e., official name change or gender designation or legal documents). This brings TNB patients in contact with many HCWs and institutions, possibly reducing meaningful interactions and diminishing patient–physician trust. 

Despite expanding literature on various barriers in healthcare, little is known about the patient–physician interactions and experiences in healthcare from TNB patients. To our knowledge, this is the first study of its kind conducted in Austria. The legal and social changes that have happened in Austria over the past decade have led to an increase in legal rights for TNB people, such as the right to change their legal gender or change their first name before any surgical interventions (a gender dysphoria diagnosis is still necessary) [28]. However, Austria has been cited by various international organizations for the lack of implementing policies that would further depathologize TNB identities [29]. To the best of our abilities, we were unable to find any studies that have provided any data on experiences of TNB people in the healthcare system in Austria. There have been sporadic results from various reports outlining that TNB patients in Austria tend to avoid accessing healthcare due to fear of prejudicial treatment by healthcare professionals and complete lack of competent and affirmative care [30,31]. These are, however, mostly anecdotal and do not provide additional insights. Given this complete lack of data on TNB experiences in healthcare from Austria and a paucity of reports from Central Europe [32], our aim was to conduct a cross-sectional study to explore the patient–physician relationship, experiences of TNB patients, transitioning facets, and health status in transgender and non-binary people in Austria. It is the hope that the results of this exploratory study serve as a base for further research highlighting the experiences of TNB people in the healthcare system in Austria, but also catalyze the needed changes to provide affirmative and competent care that TNB patients in Austria deserve. 

## 2. Participants and Methods

A cross-sectional study was carried out between June and October 2020 using a self-administered, online-based questionnaire comprising 56 items, which took about 15 min to complete, and was hosted on the SoSci Survey platform (www.soscisurvey.de, accessed on 27 June 2021) with servers based in Germany, compliant with the General Data Protection Regulation (GDPR). 

### 2.1. Participants and Recruitment

Participants in this study were recruited among individuals who were 18 years old or older, residing in Austria, and self-identifying as TNB who currently were or previously had been in the process of medical transitioning. The link to the study was distributed through various institutions and organizations involved in gender minority work, as well as through social media platforms such as Facebook, Twitter, and Instagram. This study was approved by the Ethics Committee of the Medical University of Vienna under study number 1199/2020 and was performed in accordance with the Declaration of Helsinki (1964, including subsequent revisions), as well as research guidelines developed by the European Commission. Participation in the study was voluntary, anonymous, required participants’ explicit consent, and involved no financial or any other incentives. No identifying data were saved from the participants, including the IP addresses.

### 2.2. Questionnaire

The 56 questionnaire items used in this analysis assessed sociodemographic data, gender identity and sexual orientation, aspects of medical and legal transitioning, health status and behavior, as well as experiences in healthcare settings:

1. Gender identity and satisfaction with gender expression (3 items): This initial part of the questionnaire included questions on gender assigned at birth (single-choice question: “Which sex was assigned to you at birth?” with answers being “male,” “female,” and an open-ended text entry); the participants’ current gender identity (multiple-choice question: “How would you best describe your gender identity?” with answers of “male,” “female,” “transgender (FtM, MtF),” “non-binary,” and an open-ended text entry); the satisfaction with one’s current gender expression (single-choice question: “How satisfied are you with your current gender expression?,” with answers ranging on a 5-point Likert type scale: “1 = very satisfied,” “3 = neither satisfied nor dissatisfied,” and “5 = very dissatisfied”). The questions on gender identity were based on the “two-step approach” and in line with the current recommendations of queries on gender identity in transgender and non-binary participants [33]. Additionally, prior to the study launch the questionnaire draft was presented to transgender and non-binary people to make sure the questions were understandable and non-discriminatory.
2. Sociodemographic questions and sexual orientation (15 items): Participants were asked to provide their, age, country of birth, citizenship, their current country of residence, urban characteristics of their place of residence, highest completed level of education, employment, monthly income, living situation, their sexual orientation, and degree of outness (in general and in various areas of daily life).
3. Aspects of medical and legal transitioning (14 items): Participants were asked to indicate whether they had already initiated medical transitioning and at what age, the duration of the initial psychological assessment before transitioning began, the appointed coordinator of care, various gender affirming medical procedures that they planned to have or had undergone, and whether they had legally changed their name and gender indication on official documents.
4. General experiences of violence (2 items): Participants were asked whether they have experienced violence due to their gender identity and what forms of violence they had experienced.
5. Health status and behaviors (4 items): Participants were asked to indicate whether they were ever diagnosed by a healthcare professional with a chronic illness or a mental health condition (other than receiving the “necessary” diagnosis of “gender dysphoria”), and on the frequency and amount of tobacco and alcohol they consumed.
6. Healthcare utilization and experiences in healthcare settings (9 items): Participants were asked whether they had consulted a physician over the past year and whether they would seek medical assistance again the future, whether they had ever been deliberately misgendered by a healthcare professional even after stating their pronouns, whether they had experienced any form of violence while in the healthcare setting due to their gender identity, whether they had ever been denied treatment due to their gender identity, whether they had ever reported such an incident and to whom, and their perception of the most problematic professional group within the healthcare system.
7. Doctor–patient relationship (9 items): This was assessed by the Patient-Doctor Relationship Questionnaire (PDRQ-9). The PDRQ-9 was developed from the Helping Alliance Questionnaire, which measures the therapeutic alliance in psychotherapy and provides researchers with a brief measure of therapeutic aspects of the doctor–patient relationship in primary care settings [34]. The PDRQ-9 has been shown to have high reliability and validity [34,35]. For the purposes of this study, we used the validated German-language version [36]. The PDRQ-9 comprises 9 positively worded statements on various aspect of satisfaction with the doctor–patient relationship, to which the participants indicate the appropriateness of each statement on a 5-point Likert-type scale (“1 = not at all appropriate,” “3 = appropriate,” and “5 = totally appropriate”). The final score (5–45 points) is produced as a sum of all 9 items, with higher scores indicating a better doctor–patient relationship [34]. As TNB patients in Austria have to have a primary coordinator of care to coordinate the transitioning process (which may or may not be a primary care physician), we asked the participants to answer the questionnaire with their coordinator of care in mind. In our survey, the internal consistency was determined by Cronbach’s alpha and was found to be 0.96, indicating high internal consistency.

### 2.3. Statistical Analysis

Data were analyzed with SPSS v27.0 for MacOS (Reference: IBM Corp. Released 2020. IBM SPSS Statistics for MacOS, Version 27.0. Armonk, NY: IBM Corp). All variables were analyzed descriptively, with mean values and standard deviations shown for continuous variables, and frequencies and percentages for categorical data. One-way ANOVA was performed to compare the PDRQ-9 score between the gender identity categories, including a Tukey HSD post-hoc analysis to pinpoint the group pairs with a significant difference of mean PDRQ-9 scores.

## 3. Results

The link to the online questionnaire was clicked on a total of 1280 times, and 139 participants proceeded to fill out the questionnaire. After applying the inclusion criteria (i.e., over the age of 18, residing in Austria, self-identifying as transgender or non-binary, having started medical or legal transitioning), 91 entries were eligible for analysis.

### 3.1. Sociodemographics

The majority of study participants were Austrian citizens (92.3%), with a mean age of 29 (SD = 10.0), residing in a city (68.1%), and having completed secondary education (37.4%). A total of 56.7% were in paid employment, earning up to EUR 1000/month (59.6%), and were living alone or in a shared flat (57.0%). Further descriptive variables of the sample are presented in Table 1. 

### 3.2. Identity-Related Characteristics

Seventy percent of participants were assigned female at birth and 41.8% of participants identified as non-binary, and almost one third identified as bisexual. Most reported being “somewhat” or “very” satisfied with their gender expression, and almost all were “out” (i.e., did not conceal) their gender identity and sexual orientation, as presented in Table 1.

### 3.3. Medical and Legal Transitioning

The mean age at the start of medical transitioning was 26. More than 80% were in the process of medical transitioning at the time of the study, with their psychotherapist having coordinated the referral statements. Mean waiting time for positive referral to start the medical transitioning was around 10 months. Overall, 82.4% of transitioning participants had been on hormone replacement therapy for a median duration of 35 months at the time of the survey, and 52.7% had not had surgical interventions (e.g., vaginoplasty/phalloplasty). Of those who had already undergone surgery, almost all had had one or more procedures completed in Austria. Concerning other types of therapeutic interventions, most reported psychotherapy. Regarding legal transitioning, most had changed their name and their legal gender. Details are summarized in Table 2.

### 3.4. Health Status and Behavior

More than a third of participants had a chronic disease diagnosed by a physician, and almost two thirds had a mental health diagnosis from a mental health professional. The majority never smoked, and “occasionally” consumed alcohol (Table 2).

### 3.5. Experiences in Healthcare Settings

Most participants had consulted a physician in the past year, and would “probably” seek medical attention in future for a somatic illness, whereas a higher proportion stated the same for mental illness, as shown in Table 3. Almost two thirds of participants reported not being taken seriously in medical settings, and over 20% stated being “often” and “very often” misgendered by healthcare workers (HCWs), even after stating their gender identity and pronouns. Almost 8% said they experienced verbal violence from HCWs, whereas only 4.9% reported such incidents. More than 13% reported having been denied medical care due to their gender identity, and more than half perceived physicians as the most problematic members of the healthcare workforce (Table 3). 

### 3.6. Physician–Patient Relationship

The physician-patient relationship was evaluated using the Patient-Doctor Relationship Questionnaire 9 (PDRQ-9). The mean score on this standardized questionnaire was 33.55 (SD = 8.83) out of a possible 45 points, where non-binary participants showed a lower score (M = 30.79; SD = 7.46) than trans women (M = 34.78; SD = 9.17) and trans men (M = 36.10; SD = 9.45). After a non-significant Levene statistic confirmed homogeneity of variance between these three gender identity groups, a one-way ANOVA showed a significant difference in means (F = 3.52, df = 2, *p* = 0.034). A post-hoc Tukey HSD revealed a significant difference in means between non-binary participants and trans men (Table 4).

## 4. Discussion

The findings of this exploratory cross-sectional study outline experiences and interactions in the healthcare system of TNB patients in Austria. The results present a broad scope of experiences and healthcare utilization, which are novel outcomes compared to other studies that traditionally focused on sexually transmitted illnesses or specific mental health outcomes. 

In our sample more than two thirds of participants reported being never or rarely taken seriously with regard to their gender identity in healthcare settings. Moreover, one fifth reported being very often or often misgendered by HCW. Alarmingly, 13% reported having been denied medical care due to their gender identity and 7% experienced violence. Physicians were by far the most commonly identified as the HCW group with which participants experienced the most issues. Our results echo various other reports consistently outlining that TNB people experience stigmatization and discrimination in healthcare settings. Results of the 2015 US Transgender Survey showed that more than one third of transgender individuals reported being mistreated in the past year within healthcare systems, which included various negative experiences ranging from disrespectful treatment to verbal harassment and refusal of treatment [9]. Similar results were reported in Europe, where the results of the Trans Health Survey from Georgia, Poland, Serbia, Spain, and Sweden showed that 25% of participants felt discriminated against by a healthcare provider in the past year. The most common issues were reported as lack of knowledge by 48% of participants and 42% reporting misgendering (i.e., using incorrect names or pronouns) [37]. These results are in accordance with previous research and reaffirm the need for an immediate inclusion of curriculum elements focusing on care of TNB patients [7]. Moreover, as more and more young people are identifying with more fluid concepts of gender (up to 6% of 12–17-year-olds) [38], the visibility of non-cisgender identities is rising, which will additionally bring physicians and other HCW in more direct contact with TNB patients. Practitioners from various medical fields have already voiced the need to establish guidelines and less gendered language in their respective fields [39,40]. HCW can create safe and supportive environments by using correct names and pronouns, keeping in mind the evolving nature of gender identities. Simple changes in terminology and sensitive use of language can help maintain affirmative care [41]. 

Non-binary people reported overall lower scores on the PDRQ-9 compared to trans men and trans women. Similar findings were reported by Kittari and colleagues, who reported that non-binary people were less likely to have a healthcare provider who was aware of their gender identity and treated them with respect [7]. However, another study by the same author reported that non-binary people were less likely to postpone seeking medical care due to fear of discrimination compared to trans women [42]. A possible reason may be that non-binary individuals are less likely to come out (i.e., disclose) to their healthcare providers at all [7]. Furthermore, some authors have postulated that non-binary and transgender people who identify further from the binary concept of sex (i.e., male/female) experience greater levels of discrimination. This may explain why non-binary people experienced lower scores on the PDRQ-9 score in the present findings [43]. Moreover, it outlines the need to educate HCWs and continue discussions in the medical field around the topics of diverse identities and intersectionality.

Of the participants who underwent gender-affirming surgery, 92.5% did so in Austria, and almost all were receiving hormone treatment and 74% planned to have further medical procedures in Austria. This, combined with relatively short waiting times compared with international experiences, indicates high-quality TNB care in Austria, as access to gender-affirming care is shown to have an influence on quality of life and health outcomes in TNB patients [44,45,46,47]. 

Finally, our results should be viewed in light of certain limitations. The cross-sectional study design does not allow for causal inference. Further, a sampling bias might have led to a skewed sample of participants who were more connected within the larger TNB community, with the snowballing method likely also influencing sample composition. Given the sensitive nature of some of the questions regarding experienced violence and discrimination, there may be some reporting bias whereby some participants may have felt uncomfortable indicating that they had experienced such incidents, which may have led to additional data distortion. In addition, as anecdotal evidence suggests that TNB people in Austria tend to avoid contact with the healthcare system out of fear of being judged and mistreated, it is possible that our sample overrepresented participants who were healthy and felt safe. 

However, this study is the first of its kind from Austria, providing a much-needed exploratory and descriptive analysis of experiences within and interactions with the healthcare system and healthcare professionals. While we recognize the limited sample size of this study, it is our hope that our work will serve as a foundation for larger and more comprehensive analyses of TNB patient experiences of the Austrian healthcare system and will encourage systemic changes in practice and medical professionals’ behavior to provide supportive and targeted medical care that appropriately supports these communities. 

## 5. Conclusions

The results of our exploratory study show a range of experiences that transgender and non-binary patients have in Austrian healthcare settings. The results indicate that the reported experiences are still challenging despite the growing visibility and social and legal recognitions. A high proportion of our participants reported not having access to affirming care. Comparisons of patient–physician interactions suggest that non-binary people report worse experiences, which may be explained by a lack of awareness of healthcare workers regarding evolving gender identities. Given the exploratory nature of our study, we were not able to include detailed questions of lived experiences. Therefore, qualitative and mixed-methods studies providing insights into personal experiences would be valuable and support further validity of questionnaire-based studies. Overall, more educational efforts both at medical school level and ongoing medical education, coupled with raising overall societal awareness, should be implemented to improve experiences of TNB patients in Austria. 

## Figures and Tables

**Table 1:** Sociodemographic and identity characteristics of the study sample.

Variable	Participants (*N* = 91)
Age	
M ^1^, SD ^1^	29.0 (10.0)
Austrian citizenship (*n*, %)	
No	4 (4.4%)
Yes	87 (95.6%)
Geographical area (*n*, %)	
Countryside/town	29 (31.9%)
City	62 (68.1%)
Education (*n*, %)	
None/Primary/Vocational	24 (26.4%)
Secondary	34 (37.4%)
Tertiary	33 (36.3%)
Income (*n*, %)	
Up to EUR 1000/month	53 (59.6%)
Up to EUR 2000/month	24 (27.0%)
>2000 EUR/month	12 (13.5%)
Employment status (*n*, %)	
No paid employment	39 (43.3%)
In paid employment	51 (56.7%)
Living situation (*n*, %)	
Single/Alone/Shared flat	45 (57.0%)
With partner	21 (26.6%)
Other	13 (16.5%)
Gender assigned at birth (*n*, %)	
Female	64 (70.3%)
Male	26 (28.6%)
Other	1 (1.1%)
Gender identity (*n*, %)	
Trans man	30 (33.0%)
Trans woman	23 (25.3%)
Non-binary person	38 (41.8%)
Sexual orientation (*n*, %)	
Heterosexual	10 (11.0%)
Homosexual	22 (24.2%)
Bisexual	30 (33.0%)
Asexual	3 (3.3%)
Queer/Pansexual	26 (28.6%)
Gender expression (*n*, %)	
Very dissatisfied	9 (9.9%)
Somewhat dissatisfied	10 (11.0%)
Neutral	10 (11.0%)
Somewhat satisfied	41 (45.1%)
Very satisfied	21 (23.1%)
Out (*n*, %)	
Yes	90 (98.9%)
No	1 (1.1%)

^1^ M = mean value (arithmetic mean), SD = standard deviation.

**Table 2:** Aspects of transitioning and health-related characteristics.

Variable	Participants (*N* = 91)
Medically transitioning (*n*, %)	
No	16 (17.6%)
Yes	75 (82.4%)
* Age at begin of transitioning	
M (SD)	26.2 (8.9)
* Coordinator of referral statement (*n*, %)	
Psychotherapist	55 (75.3%)
Psychologist	4 (5.5%)
Psychiatrist	6 (8.2%)
Gynecologist	1 (1.4%)
Endocrinologist	1 (1.4%)
Other	6 (8.2%)
* Duration of wait for positive referral	
M (SD)	10.2 (7.7)
* Hormonal replacement therapy (HRT) (*n*, %)	
No	9 (12.3%)
Yes	64 (87.7%)
* Months on HRT	
M (SD)	35.5 (38.5)
* Gender-affirming surgery (*n*, %)	
Chest (top)	20 (27.0%)
Other (±top)	15 (20.3%)
None	39 (52.7%)
** Surgery abroad (*n*, %)	
Yes	2 (7.4%)
No	25 (92.6%)
* Speech therapy (*n*, %)	
No	52 (74.3%)
Yes	15 (21.4%)
* Epilation (*n*, %)	
No	53 (79.1%)
Yes	14 (20.9%)
* Psychotherapy (beyond the scope for referrals) (*n*, %)	
No	22 (32.8%)
Yes	45 (67.2%)
Further medical steps desired (*n*, %)	
No	37 (41.6%)
Yes	52 (58.4%)
Location of potential further medical steps (*n*, %)	
Austria	48 (73.8%)
Abroad	17 (26.2%)
Legal name change (*n*, %)	
Yes	69 (75.8%)
No, but planned	19 (20.9%)
No, not planned	3 (3.3%)
Legal gender change (*n*, %)	
Yes	59 (64.8%)
No, but planned	27 (29.7%)
No, not planned	5 (5.5%)
Chronic somatic illness (*n*, %)	
Yes	32 (35.2%)
No	59 (64.8%)
Mental illness (n, %)	
Yes	52 (57.1%)
No	39 (42.9%)
Smoking status (*n*, %)	
Yes	11 (12.1%)
No, ex-smoker	33 (36.3%)
Never smoked	47 (51.6%)
Alcohol consumption (*n*, %)	
Never	19 (20.9%)
Occasionally	58 (63.7%)
Weekly	9 (9.9%)
Daily	5 (5.5%)

* Considering only participants who were medically transitioning (*n* = 75). ** Considering only participants who underwent gender-affirming surgery (*n* = 35).

**Table 3:** Experiences in healthcare settings.

Variable	Participants (*N* = 91)
Consulted a physician in the past year (*n*, %)	
Yes	69 (75.8%)
No	22 (24.2%)
Planned somatic healthcare utilization, if needed (*n*, %)	
Definitely not	0 (0.0%)
Probably not	4 (4.4%)
Maybe	14 (15.4%)
Probably yes	37 (40.7%)
Definitely yes	36 (39.6%)
Planned mental healthcare utilization, if needed (*n*, %)	
Definitely not	3 (3.3%)
Probably not	4 (4.4%)
Maybe	12 (13.2%)
Probably yes	18 (19.8%)
Definitely yes	54 (59.3%)
Taken seriously in healthcare setting	
Never	34 (37.4%)
Rarely	21 (23.1%)
Sometimes	19 (20.9%)
Often	11 (12.1%)
Very often	6 (6.6%)
Misgendered by healthcare workers (HCW)	
Never	37 (41.1%)
Rarely	17 (18.9%)
Sometimes	17 (18.9%)
Often	10 (11.1%)
Very often	9 (10.0%)
Violence by HCW	
Yes	7 (7.7%)
No	84 (92.3%)
Denial by HCW	
Yes	12 (13.2%)
No	79 (86.8%)
Reported incident with HCW	
Yes	2 (4.9%)
No	39 (95.1%)
Subjectively most problematic HCW	
Physicians	36 (58.1%)
Nurses	6 (9.7%)
Medical–technical personnel	3 (4.8%)
Psychologists	11 (17.7%)
Speech therapists	0 (0.0%)
Other	6 (9.7%)

**Table 4:** Tukey HSD post-hoc correction of ANOVA comparing mean PDRQ-9 scores of different gender identity categories.

Gender Identity Group (A)	Gender Identity Group (B)	Mean Difference (A − B)	*p*	Cohen’s d (SMD *)	95% Confidence Interval (Lower Bound, Upper Bound)
Trans men	Trans women	1.32	0.85	0.15	−4.36, 7.00
	Non-binary	5.31	0.04	0.60	0.31, 10.32
Trans women	Trans men	−1.32	0.85	−0.15	−7.00, 4.36
	Non-binary	3.99	0.19	0.45	−1.42, 9.41
Non-binary	Trans men	−5.31	0.04	−0.60	−10.32, −0.31
	Trans women	−3.99	0.19	−0.45	−9.41, 1.42

* SMD = standardized mean difference.

## Data Availability

Limited dataset is available upon reasonable request from the corresponding author.
